# Pulmonary strongyloidiasis: assessment between manifestation and radiological findings in 16 severe strongyloidiasis cases

**DOI:** 10.1186/s12879-017-2430-9

**Published:** 2017-05-02

**Authors:** Daijiro Nabeya, Shusaku Haranaga, Gretchen Lynn Parrott, Takeshi Kinjo, Saifun Nahar, Teruhisa Tanaka, Tetsuo Hirata, Akira Hokama, Masao Tateyama, Jiro Fujita

**Affiliations:** Department of Infectious Diseases, Respiratory, and Digestive Medicine, Graduate School of Medicine, University of the Ryukyus, 207 Uehara, Nishihara, Okinawa, 903-0215 Japan

**Keywords:** Acute respiratory distress syndrome, Bacterial pneumonia, Interlobular septal thickening, Pulmonary alveolar hemorrhage, pulmonary strongyloidiasis

## Abstract

**Background:**

Strongyloidiasis is a chronic parasitic infection caused by *Strongyloides stercoralis.* Severe cases such as, hyperinfection syndrome (HS) and disseminated strongyloidiasis (DS), can involve pulmonary manifestations. These manifestations frequently aid the diagnosis of strongyloidiasis. Here, we present the pulmonary manifestations and radiological findings of severe strongyloidiasis.

**Methods:**

From January 2004 to December 2014, all patients diagnosed with severe strongyloidiasis at the University of the Ryukyus Hospital or affiliated hospitals in Okinawa, Japan, were included in this retrospective study. All diagnoses were confirmed by the microscopic or histopathological identification of larvae. Severe strongyloidiasis was defined by the presence of any of the following: 1) the identification of *S. stercoralis* from extra gastrointestinal specimens, 2) sepsis, 3) meningitis, 4) acute respiratory failure, or 5) respiratory tract hemorrhage. Patients were assigned to either HS or DS. Medical records were further reviewed to extract related clinical features and radiological findings.

**Results:**

Sixteen severe strongyloidiasis cases were included. Of those, fifteen cases had pulmonary manifestations, eight had acute respiratory distress syndrome (ARDS) (53%), seven had enteric bacterial pneumonia (46%) and five had pulmonary hemorrhage (33%). Acute respiratory failure was a common indicator for pulmonary manifestation (87%). Chest X-ray findings frequently showed diffuse shadows (71%). Additionally, ileum gas was detected for ten of the sixteen cases in the upper abdomen during assessment with chest X-ray. While, chest CT findings frequently showed ground-glass opacity (GGO) in 89% of patients. Interlobular septal thickening was also frequently shown (67%), always accompanying GGO in upper lobes.

**Conclusions:**

In summary, our study described HS/DS cases with pulmonary manifestations including, ARDS, bacterial pneumonia and pulmonary hemorrhage. Chest X-ray findings in HS/DS cases frequently showed diffuse shadows, and the combination of GGO and interlobular septal thickening in chest CT was common in HS/DS, regardless of accompanying pulmonary manifestations. This CT finding suggests alveolar hemorrhage could be used as a potential marker indicating the transition from latent to symptomatic state. Respiratory specimens are especially useful for detecting larvae in cases of HS/DS.

## Background

Strongyloidiasis is a chronic parasitic infection caused by *Strongyloides stercoralis*. Strongyloidiasis is occasionally recognized as a “neglected tropical disease” [1–4]. Previous reports have estimated 30 to 100 million infected persons worldwide [5–7], with some reports predicting a high likelihood of increase [8]. However, due to increasing globalization and immigration, the development of symptomatic strongyloidiasis and diagnosis of strongyloidiasis may become problematic for non-endemic areas [9, 10]. As such, there is a chance for symptomatic patients with strongyloidiasis living in non-endemic countries to spread the disease exponentially.

**Fig. 1:**
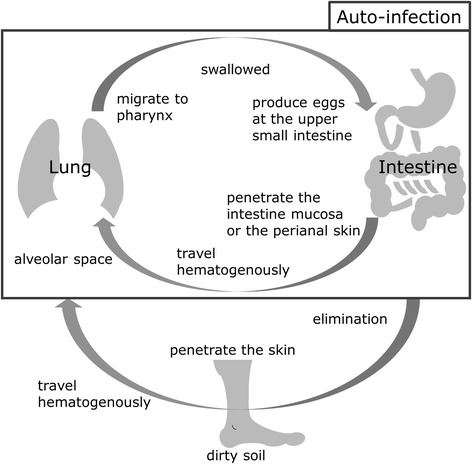
Lifecycle of *Strongyloides stercoralis*

This parasite has unique life cycle (Fig. 1). Filariform larvae, which inhabit the soil, infect humans via skin penetration. After infection, the larvae travel hematogenously to the lung and then escape to alveolar space. The larvae then migrate to the pharynx and are swallowed, producing eggs in the upper small intestine. Rhabditiform larvae, hatched from eggs, are usually excreted from the human host. However, some rhabditiform larvae can mature into filariform larvae within the bowel, and re-infect their host via the intestinal mucosa or perianal skin. This re-infection process, called auto-infection, allows *S. stercoralis* to complete its life cycle and proliferate successfully within a single host [7, 11, 12]. As such, it is possible for *S. stercoralis* to infect a host for years or decades without detection [13].

The majority of infected patients are asymptomatic or present only mild gastrointestinal symptoms [10, 14–16]. However, some patients, such as those with advanced age, malnutrition, those using an immunosuppressant including steroids, or those with diabetes mellitus and human T-cell leukemia virus type 1 (HTLV-1) can progress to hyperinfection syndrome (HS), characterized by complications due to uncontrolled proliferation of larvae, and disseminated strongyloidiasis (DS), characterized by disseminating to organs not involved in the life-cycle of *S. stercoralis* in humans [7, 14, 15, 17–20]. Because patients with HS/DS are often at risk for further complications like sepsis and/or meningitis, the fatalities within this group are common, ranging from 60 to 80% [9, 17, 21]. Interestingly, HS/DS can also involve pulmonary manifestations [17, 22–24]. Often called, pulmonary strongyloidiasis, this manifestation can facilitate the diagnosis of strongyloidiasis. Although some reports discuss radiological findings using chest X-ray [17, 25], no published data exists to our knowledge comparing pulmonary manifestations with the radiological findings of chest CT in severe strongyloidiasis cases.

In an effort to lessen this knowledge gap, we present the pulmonary manifestations for sixteen cases of severe strongyloidiasis from Okinawa, Japan, a subtropical region previously considered endemic for *S. stercoralis* [26, 27].

### Methods

From January 2004 to December 2014, all patients diagnosed with severe strongyloidiasis at the University of the Ryukyus Hospital or affiliated hospitals in Okinawa, Japan, were included in this retrospective study. All diagnoses were confirmed by the microscopic identification of larvae using the agar plate culture method [11] or histopathological findings. For purposes of this study, severe strongyloidiasis was defined by the presence of any of the following: 1) the identification of *S. stercoralis* from extra gastrointestinal specimens, 2) sepsis, 3) meningitis, 4) acute respiratory failure, or 5) respiratory tract hemorrhage. Severe cases were assigned to DS when larvae were found in any organ, other than those within the respiratory and gastrointestinal tracts (organs not involved in the life-cycle of *S. stercoralis* in humans). Other cases were assigned to HS. Medical records were reviewed to extract any relevant and related clinical features.

Radiological findings of chest X-ray (CXR) and chest computed tomography (CCT) taken at the onset of HS/DS were also reviewed. If CXR or CCT was performed multiple times, an image from the date closest to onset was chosen for radiological assessment. All radiological findings were reviewed by two respirologists, and two gastroenterologists checked for the existence of ileum gas using the same CXR. Patients with a simultaneous underlying pulmonary disease were excluded from the analysis of radiological findings, due to difficulties in identifying the root cause of radiological findings.

The study was reviewed and approved by the Clinical Research Ethics Committee of University of the Ryukyus (H26.8–7-695).

## Results

### Patient characteristics

In total, sixteen severe strongyloidiasis cases were collected: three DS and thirteen HS (Tables 1, 2). Fourteen cases had detectable *S. stercoralis* in extra-gastrointestinal specimens. Of importance, thirteen of those fourteen cases could be detected from respiratory specimen. Thirteen cases had gastrointestinal complications and ten had sepsis and/or meningitis. HTLV-1 infection and hypoalbuminemia (<3.8 mg/dL) were the most common patient characteristics. Five cases died within thirty days after diagnosis of strongyloidiasis (case fatality rate 31%). Patient background and complications were compared between the five fatalities and all other cases in Table 3. All fatal cases had pulmonary complications and systemic infection. The findings for case number 1 and 2 have been previously published as case report articles [28] (the report for case 1 was published as a Japanese article).

**Table 1:** Patient and Sample Charactaristics

*n* = 16		(%)
Male	7	(44)
Age^a^	75	(46–96)
Underlying conditions		
Serum albumin (g/dL)^ab^	2.3	(1.1–4.2)
HTLV-1^c^	11	(79)
Steroid user	5	(31)
Chemotherapy	1	(6)
Underlying disease		
Adult T-cell lymphoma/leukemia	5	(31)
Type 2 diabetes mellitus	3	(19)
Chronic heart disease	3	(19)
Cervical cancer	1	(6)
Rheumatoid arthritis	1	(6)
Pulmonary disease	2	(12)
Chronic obstructive pulmonary disease	2	
Lung cancer	1	
Interstitial pneumonia	1	
Sample type		
Respiratory	13	(81)
Sputum	11	
Bronchoscopic lavage	2	
Gastro-intestine	13	(81)
Stool	11	
Gastric juice	6	
Gastric biopsy	1	
Out of life cycle organ (DS)	3	(19)
Urine	2	
Ascites	1	

Abbreviations: *DS* disseminated strongyloidiasis, *HTLV-1* human T-cell leukemia virus type 1, ^a^mean (range) was used for these values, ^b^total 13 cases were tested serum albumin, ^c^total 14 cases were tested HTLV-1

**Table 2:** Clinical information

*n* = 16		(%)
Cases with systemic infection	10	(63)
Sepsis	8	
(3 *Klebsiella pneumoniae*, 2 *Escherichia coli*, 1 *Aeromonas hydrophila*, 2 unknown pathogen)		
Meningitis	5	
(1 *Klebsiella pneumoniae*, 1 *Escherichia coli*, 3 unknown pathogen)		
Cases with gastro-intestinal complication	13	(81)
Vomiting	9	
Diarrhea	5	
Ileus	4	
Constipation	3	
Abdominal pain	2	
Ascites	2	
Melena	1	
Treatment		
Ivermectin	16	(100)
daily	12	
weekly	1	
+ Thiabendezole	1	
regimen is not clear	3	
Died in 30 days from diagnosis	5	(31)

**Table 3:** Comparison between survivors and non-survivors

	Non-survivors		Survivors	
	*n* = 5	(%)	*n* = 11	(%)
Male	1	(20)	6	(55)
Age^a^	66.8	(46–81)	79.8	(51–96)
Underlying conditions				
Serum albumin (g/dL)^ab^	2.0	(1.5–2.6)	2.4	(1.1–4.2)
HTLV-1^c^	3	(75)	8	(80)
Steroid user	2	(40)	3	(27)
Anti-cancer drug	0		1	
Underlying disease				
Adult T-cell lymphoma/leukemia	1	(20)	4	(27)
Type 2 diabetes mellitus	2	(40)	1	(9)
Cases with pulmonary complication	5	(100)	10	(91)
Acute respiratory distress syndrome	4		4	
Pulmonary alveolar hemorrhage	1		2	
Other complication				
Systemic infection	5	(100)	5	(45)
Sepsis	4		4	
Meningitis	3		2	
Ileus	1	(20)	3	(27)

Abbreviations: HTLV-1 = human T-cell leukemia virus type 1, ^a^mean (range) was used for these values, ^b^total 13 cases (3 non-survivors and 10 survivors) were tested serum albumin, ^c^total 14 cases (4 non-survivors and 10 survivors) were tested HTLV-1

### Pulmonary manifestations

Meanwhile, fifteen of the sixteen cases had pulmonary manifestations (Table 4). Acute respiratory distress syndrome (ARDS) was the most common manifestation (8/15), bacterial pneumonia (7/15) and respiratory hemorrhage including pulmonary alveolar hemorrhage (PAH) and hemoptysis (5/15) followed. Acute respiratory failure (ARF) was a common indication for pulmonary manifestations (13/15). In cases with bacterial pneumonia, pathogens detected were always enteric bacteria; 2 *Klebsiella pneumoniae*, 1 *Escherichia coli*, 1 *Acinetobacter baumannii*, 1 *Citrobacter koseri* (all pathogens were identified from sputum culture).

**Table 4:** Pulmonary manifestations

Pulmonary Manifestations		
*n* = 16		(%)
Cases with pulmonary complication	15	(94)
Acute respiratory failure	13	
Acute respiratory distress syndrome	8	
Bacterial pneumonia	7	
Hemorrhage		
Pulmonary alveolar hemorrhage	3	
Hemoptysis	2	
Acute exacerbation of interstitial pneumonia	1	

### Radiological Findings

**Fig. 2:**
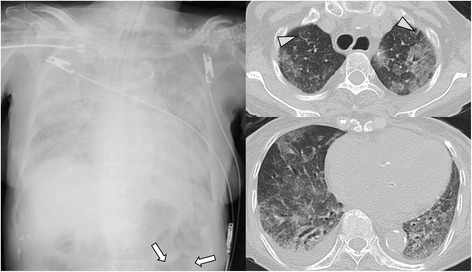
Case 3: A patient with larvae detected from respiratory specimen, received steroid therapy and developed sepsis with acute respiratory failure (hyperinfection syndrome). Broncho-alveolar lavage revealed pulmonary alveolar hemorrhage and larvae. Chest X-ray shows diffuse consolidation and ileum gas (arrow). CT shows diffuse ground-glass opacity with slight inter-lobular septal thickening (arrow head) and pleural effusion

**Fig. 3:**
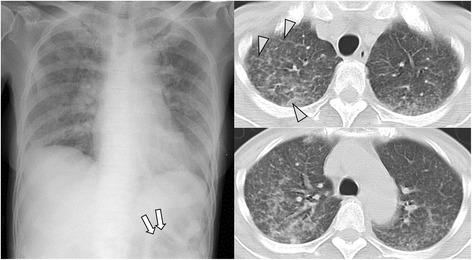
Case 4: A patient with larvae detected from respiratory specimen, received steroid therapy and developed pneumonia, sepsis and meningitis with hemoptysis and acute respiratory distress syndrome (hyperinfection syndrome). Chest X-ray shows diffuse ground-glass opacity and ileum gas (arrow). CT shows multi-focal ground-glass opacity with slight inter-lobular septal thickening (arrow head)

**Fig. 4:**
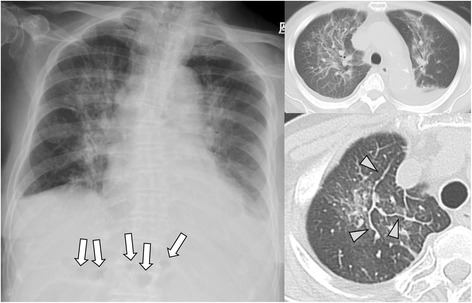
Case 15: A adult T-cell lymphoma patient with larvae detected from intestinal specimen, developed pneumonia and meningitis with acute respiratory failure (hyperinfection syndrome). Chest X-ray shows diffuse consolidation and ileum gas (arrow). CT shows diffuse ground-glass opacity and consolidation with inter-lobular septal thickening (arrow head), broncho-vascular bundle thickening and pleural effusion

**Fig. 5:**
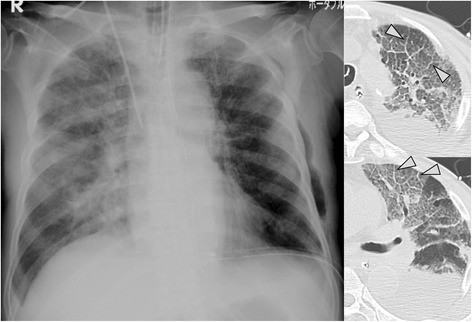
Case 16: A patient with larvae detected from respiratory specimen, received steroid therapy and developed sepsis with acute respiratory distress syndrome (hyperinfection syndrome). Chest X-ray shows diffuse ground-glass opacity and consolidation in the lung, as well as a chest drainage tube in the lower left thoracic cavity. Ileum gas could not be detected. CT shows diffuse ground-glass opacity and obvious inter-lobular septal thickening (arrow head), so-called crazy-paving, and pleural fluid

For this assessment, two cases (case 8 and 12) were excluded because pulmonary strongyloidiasis findings were difficult to distinguish from underlying lung diseases; severe emphysema and interstitial pneumonia (Table 5). CXR was assessed in the remaining fourteen cases and thirteen cases had findings. Typical findings for pulmonary strongyloidiasis are shown (Figs. 2, 3, 4, 5). Ten of the thirteen cases with findings had diffuse shadow, and of those ten, four cases had opacities beginning at the hilum radiating upward to the middle sternum, like butterfly pulmonary opacity (i.e. Fig. 4). Another three cases had focal shadows. Additionally, ileum gas was detectable for ten of sixteen cases in the upper abdomen during assessment with CXR and nine of these cases had ileum gas present in the upper left abdomen.

**Table 5:** Chest Radiological findings

X-ray			CT		
*n* = 14		(%)	*n* = 9		(%)
Diffuse			Diffuse		
GGO	4	(29)	GGO	3	(33)
Consolidation	4	(29)	GGO ~ Consolidation	3	(33)
GGO ~ Consolidation	2	(14)	Multi-focal		
Multi-focal GGO	1	(7)	GGO	1	(11)
Focal			Consolidation	1	(11)
GGO	1	(7)	Focal GGO	1	(11)
Consolidation	1	(7)			
No abnormalities in lung	1	(7)	Broncho-vascular bundle thickening	2	(22)
Costophrenic angle dull	2	(14)	Inter-lobular septal thickening^a^	6	(67)
Ileum gas in upper abdomen	10	(71)	Pleural fluid	8	(89)

Abbreviation: *GGO* ground glass opacity, ^a^always accompanied GGO in upper lobes

CCT findings were assessed in nine cases. Eight of those nine cases had ground-glass opacity (GGO), and six had diffuse GGO primarily in the upper lobes (i.e. Figs. 4 and 5). The combination of GGO with interlobular septal (ILS) thickening was also a common radiologic finding (6/9). Cases 2 [28] and 16 (Fig. 5) exemplified ILS thickening with GGO, with so called crazy-paving appearance. Almost all cases (8/9) had pleural fluid.

## Discussion

There is much diversity among the manifestations of pulmonary strongyloidiasis. However, it is likely most patients present with one or more of the three most common complications; bacterial pneumonia, alveolar hemorrhage and allergic/eosinophilic manifestation from larvae [17, 22, 23, 25, 29, 30]. In patients with HS/DS, the number of larvae in the host is continuously multiplying [12, 31]. As this happens, many larvae pass through the alveolar membranes causing, sometimes, immense tissue damage. This process is the assumed cause of bacterial pneumonia and alveolar hemorrhage in HS/DS [17, 22].

In HS/DS, enteric bacteria may gain access to the bloodstream, lung and other organs throughout the body with the filariform larvae. This transmission pathway is considered likely in cases of sepsis, meningitis and pneumonia by enteric bacteria co-infecting patients with HS/DS [32, 33]. As possible proof of this hypothesis, all patients’ bacterial pneumonia in our study had enteric bacteria as the causative pathogen.

Strongyloidiasis is considered one of the causes of infectious diffuse alveolar hemorrhage [30]. Some reports show autopsy revealed latent alveolar hemorrhages for mortal cases of acute respiratory failure with strongyloidiasis [29, 34]. Therefore, it can be assumed that alveolar hemorrhage due to strongyloidiasis does not always present with symptomatic hemoptysis.

In our cohort, almost all cases experienced more pulmonary manifestations than gastro-intestinal manifestations, and ARDS, bacterial pneumonia and pulmonary hemorrhage were common. This is compatible with past reports [17, 22, 23, 25]. No cases experienced allergic/eosinophilic pulmonary manifestations in our study. It is thought that eosinophilic reactions are often suppressed or absent in HS/DS due to concomitant bacterial infection, immunosuppressant like steroids or HTLV-1 infection [14, 19, 23, 35–37]. Therefore, pulmonary manifestations resulting from allergic/eosinophilic stimulation may be not common in severe HS/DS.

In chronic, uncomplicated cases of strongyloidiasis, the larvae can only be detected in a gastro-intestinal specimen. However, in HS/DS cases, the larvae of *S. stercoralis* continuously multiply within a single host, as mentioned above. Therefore, the larvae are often detected within respiratory specimens [23, 38]. Not surprisingly, almost all cases in our study had discernable larvae within their respiratory specimens. Therefore, we suggest that analysis of the respiratory specimen is a convenient and useful technique for the diagnosis of strongyloidiasis in HS/DS.

In our HS/DS cases, low serum albumin and HTLV-1 were common and this agrees with other reports [14, 15, 19]. In patients with HTLV-1 and *S. stercoralis* co-infection, regulatory T-cell counts are increased and correlate with both low circulating eosinophil counts and reduced antigen-driven IL-5 production [19]. Low serum albumin has not been confirmed as a factor driving HS/DS or the result of long term infection.

All five fatal cases had systemic bacterial infection. However, the total rate of concomitant bacterial infection was not so different from past reports [23, 39]. Additionally, the number of deaths in our study was low compared with past reports [9, 17, 21]. It is possible different treatment regimens could explain this difference. Ivermectin is currently the gold standard for the treatment of strongyloidiasis, and ivermectin was used for all cases in our study. In fact, a systematic review showed cases treated with albendezole or thiabendazole had an increased percentage of deaths among patients than cases treated with ivermectin [9]. Seggarra-Newnham recommend that treatment for HS/DS is to administer ivermectin daily until symptoms resolve and stool tests have been negative for at least two weeks [7]. The research Group on Chemotherapy of Tropical Disease, Japan, also recommends HS/DS cases complicated with acute respiratory failure commonly use daily ivermectin until the disappearance of larvae in both sputum and stool. However, these recommendations do not have an evidence-based basis.

ILS thickening with GGO was seen often in our study, and some cases had the appearance of crazy-paving, as a characteristic finding of alveolar hemorrhage. Considering strongyloidiasis is one cause of infectious alveolar hemorrhage [30], it is assumed these findings could indicate alveolar hemorrhage as a marker for the transition stage from the latent to the symptomatic state.

CXR findings of diffuse shadows, like butterfly pulmonary opacities, are also compatible with pulmonary edema. Therefore it is possible, HS/DS cases with ARF and diffuse shadow could be mis-diagnosed as ARDS. ARDS cases with sepsis or meningitis, contracted in an endemic area of *S. stercoralis*, should be checked for the presence of larvae in gastro-intestinal and respiratory specimens.

Interestingly, more than half of our cases had detectable ileum gas in the upper left abdomen of CXR images. Paralytic ileus complicated with strongyloidiasis frequently occurs at the upper small intestine, because eggs deposited in the intestinal mucosa, hatch and migrate to the lumen to mature here. Therefore, ileum gas usually presents at upper left abdomen (end of duodenum to upper small intestine) [40–44], and results in our study were compatible. Although this finding deviates from the primary subject of our study, it is possible ileum gas may be an additional indicator for the presence of *S. stercoralis* when combined with other diagnostic factors.

Our study has certain limitations. First, this is retrospective study, therefore information regarding manifestations was limited. Additionally, radiological findings, taken at the time of diagnosis, were not performed with standardized procedures, conditions and timing. Lastly, we included only severe cases of strongyloidiasis, in our study. Pulmonary involvement is also present in chronic strongyloidiasis with mild/no symptoms, although the radiological manifestations might differ [45, 46]. Mild cases may also include pulmonary manifestations resulting from an allergic/eosinophilic response [17, 22].

## Conclusions

In summary, our study describes HS/DS cases with pulmonary manifestations including, ARDS, bacterial pneumonia and pulmonary hemorrhage. CXR findings in HS/DS frequently showed diffuse shadows. CCT findings revealed that GGO with ILS thickening was common in HS/DS, regardless of accompanying pulmonary manifestations. This CCT finding suggests alveolar hemorrhage could be used as a potential marker indicating the transition from latent to symptomatic state. Respiratory specimens are especially useful for detecting larvae in cases of HS/DS.

## Abbreviations

ARDS: Acute respiratory distress syndrome
avg.: Average
BBB: Broncho-vascular bundle
CCT: Chest computed tomography
CXR: Chest X-ray
GGO: Ground glass opacity
HS/DS: Hyperinfection syndrome and disseminated strongyloidiasis
HTLV-1: Human T-cell leukemia virus type 1
ILS: Interlobular septal
PAH: Pulmonary alveolar hemorrhage
